# Sentinel laboratory compliance with best practices in Burkina Faso’s antimicrobial resistance surveillance network

**DOI:** 10.4102/ajlm.v13i1.2259

**Published:** 2024-01-30

**Authors:** Dame Yenyetou, Emmanuel Zongo, Emilie Dama, Merci Muhigwa, Issouf Sanou, Charles Sawadogo, Soumaya Ouangraoua, Ibrahim Sangare, Abdoulaye Nikiema, Anicet G. Dahourou, Abdoul S. Ouedraogo

**Affiliations:** 1 Laboratoire National de Référence des Résistances aux Antimicrobiens, Centre Hospitalier Universitaire Souro SANOU, Bobo-Dioulasso, Burkina Faso; 2 Laboratoire des Pathogènes Emergents et Reémergents (LaPathER), École Doctorale Sciences de la Santé, Université Nazi BONI, Bobo-Dioulasso, Burkina Faso; 3 Institut de Recherche en Sciences de la Santé, Ouagadougou, Burkina Faso; 4 US Centers for Disease Control and Prevention, Ouagadougou, Burkina Faso; 5 Service des Systèmes d’Information, et de l’Évaluation de la Qualité, Centre Muraz, Ouagadougou, Burkina Faso; 6 Direction des Laboratoires de Biologie Médicale, Ministère de la Santé, Ouagadougou, Burkina Faso; 7 Jhpiego Burkina Faso office, Ouagadougou, Burkina Faso; 8 Integrated Quality Laboratory Services, Ouagadougou, Burkina Faso

**Keywords:** culture media, antibiotic susceptibility tests, quality control, sentinel laboratories, Burkina Faso

## Abstract

**Background:**

Standardising procedures is the best way to harmonise and strengthen the quality of laboratory-based antimicrobial resistance surveillance. Since 2018, Burkina Faso has developed and disseminated the national manual of procedures for performing antibiotic susceptibility tests in sentinel laboratories within its national antimicrobial resistance surveillance network.

**Objective:**

Our study aimed to assess these sentinel laboratories’ compliance with good practices for antibiotics susceptibility tests.

**Methods:**

Four teams evaluated the antimicrobial resistance sentinel sites laboratories throughout Burkina Faso from 19 to 28 September 2022. Eighteen out of 19 sentinel laboratories were evaluated. A four-member technical committee designed and validated the evaluation tool composed of three Microsoft Excel sheets. The evaluation emphasised quality controls for culture media, antibiotic discs and compliance with antimicrobial susceptibility testing procedures by the laboratories. Excel software was used for data recording and graphs and table design. The free R software version 4.2.0 was used for descriptive statistics. An overall score below 80% was considered noncompliance.

**Results:**

Most (83.33%) of the sentinel laboratories conducted at least one quality control activity for culture media, and 66.67% conducted at least one quality control activity for antibiotic discs. Over three-quarters (76.47%) of the laboratories were more than 80% compliant with the modified Kirby Bauer antimicrobial susceptibility testing method.

**Conclusion:**

The evaluation revealed the noncompliance of sentinel laboratories with the national procedure manual, particularly in the quality control component.

**What this study adds:**

This study has provided baseline data on the sentinel laboratories’ compliance with the national antimicrobial susceptibility testing procedures manual, particularly in areas performing quality control checks or meeting quality indicators for culture media and antibiotic discs.

## Introduction

Antimicrobial susceptibility testing (AST) allows for appropriate antibiotic therapy and monitoring of bacterial antibiotic resistance that threatens our ability to treat potentially fatal infections.^1,2^ For monitoring to happen, susceptibility testing results must be of good quality. However, in Africa, the quality of microbiological results on antimicrobial resistance (AMR) is a significant challenge.^3^ Indeed, very unusual results combinations have been found in several African studies. For instance, a study conducted in Egypt revealed that over 80% of *Proteus* isolates were resistant to imipenem, 80% were sensitive to ceftriaxone, and 50% were sensitive to vancomycin.^4^ Other studies in Ethiopia, Namibia, and Rwanda have shown resistance to penicillin in *Streptococcus pyogenes* and vancomycin resistance in *Staphylococcus aureus* isolates from cerebrospinal fluid.^5,6,7^ The susceptibility test result quality depends on the equipment (antibiotic discs and culture media), compliance with standard operating procedures, and good laboratory practices.^8^

To improve data quality, the World Health Organization encourages countries to develop a national action plan to tackle AMR.^1^ One of the priorities of this national action plan is the implementation of laboratory-based surveillance for AMR. Establishing a laboratory network under the coordination of a reference laboratory is essential to provide factual data to estimate the impact and evolution of AMR in a country. The quality of the data collected from laboratories is one of the significant challenges of this surveillance. The quality of the data collected depends on the availability of qualified human resources, appropriate equipment, and suitable methods. Harmonisation of practices and use of standardised procedures improve the quality of the data collection and correct interpretation. They also allow inter-laboratory data comparison and sharing at several levels (national, regional, and international) through the data entry and processing software provided by World Health Organization.^9^

In 2017, Burkina Faso’s Ministry of Health developed an AMR national action plan, which included the implementation of laboratory-based surveillance of AMR.^10^ As part of the Global Health Security Agenda programme, the African Society for Laboratory Medicine, with financial and technical support from the United States Centers for Disease Control and Prevention, supported the Ministry of Health in establishing the laboratory-based surveillance system of AMR in 2018. The overarching goal of the system is to provide comparable and validated data on the prevalence and trends of AMR in a group of bacteria of interest for both the World Health Organization Global Antimicrobial Resistance Surveillance System and the country.^11^ A national guideline for laboratory-based surveillance of AMR and a standardised national AST procedure was developed and endorsed by the Ministry of Health^12^ to support the implementation of this surveillance system. To ensure the accuracy and reliability of susceptibility testing results, the quality of the reagents used, and the competence of the staff performing and interpreting the tests, the guideline required laboratories to carry out weekly quality control of antibiotic discs and the culture media after preparation.^13,14^ However, no study data on quality control of sentinel laboratories for AMR have been found since implementing laboratory surveillance in Burkina Faso. To ensure compliance with the national AST procedure and the quality of data produced by sentinel laboratories, the National Antimicrobial Resistance Surveillance Laboratory, with support from the United States Centers for Disease Control and Prevention through the African Society for Laboratory Medicine and Johns Hopkins Program for International Education in Genecology and Obstetrics (Jhpiego), initiated this first quality control study of sentinel AMR laboratories. This study aimed to assess the compliance of sentinel laboratory practices with the national AST procedure and propose an action plan to correct non-conformities.

## Methods

### Ethical considerations

Ethical approval was obtained from the Burkina Faso Ministry of Health (No. 004725MS/SG/DGAP/DLBM/Dmp – 24 November 2021). This study did not involve human subjects or animal research. All the methods and procedures carried out in this study were in accordance with the Declaration of Helsinki and adhered to Burkina Faso national health research and ethics guidelines. All participating laboratories received detailed information about the study, including the purpose of the assessment and the audit plan.

### Laboratories included

**FIGURE 1:**
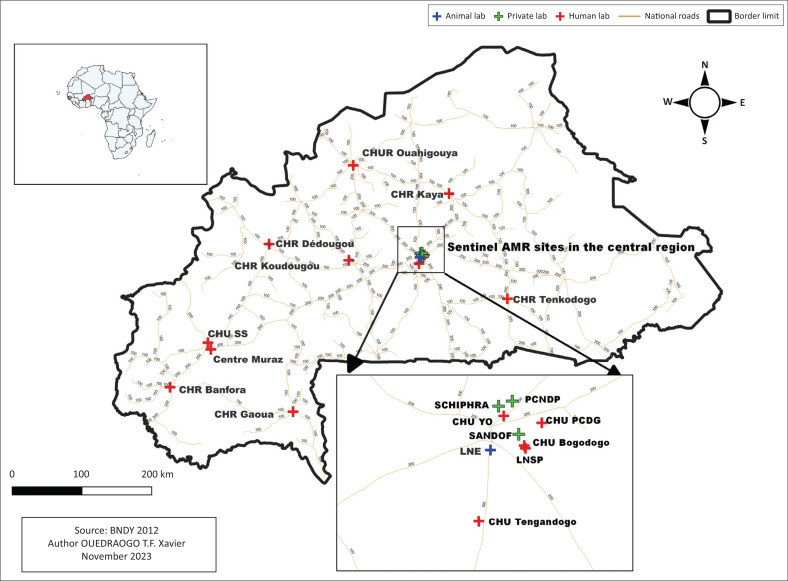
Location of the evaluated antimicrobial resistance sentinel sites in Burkina Faso, 2022.

Eighteen AMR surveillance sentinel site laboratories across Burkina Faso were evaluated (Figure 1). Sentinel site laboratories were selected based on their human (biomedical technologists and biologists) and material resources to effectively detect resistance using standardised methods and their geographical distribution to cover the whole country. There were three private laboratories and 15 public laboratories: eight from the district level and seven from the intermediate level.

### Development and validation of assessment tool

A technical committee developed and validated the assessment tool. The committee is composed of representatives from the Department of Medical Biology Laboratories of the Ministry of Health, an international expert in the evaluation of biology laboratories, specialists in quality management and microbiologists from the National Antimicrobial Resistance Surveillance Laboratory. The committee reviewed the existing literature on AST and quality control to select the indicators for the assessment tool.^11,12,15^

The assessment tool was composed of three Excel sheets. The first sheet contained four indicators on the performance of quality control of culture media (control of the pH, measuring the thickness of the culture medium, checking culture media fertility, and sterility). The second sheet had seven indicators on the presence of reference strains and the performance of quality control of the antibiotic discs (having *Escherichia coli* ATCC 25922 (CIP 7624), *Staphylococcus aureus* ATCC 25923 (CIP 7625), and *Pseudomonas aeruginosa* ATCC 27853 (CIP 76110), monitoring the condition of desiccants, defining the periodicity of quality control of the discs and the number of quality controls carried out over a given period, and verifying if the diameters of the inhibition zones are checked). The last sheet included the stages of carrying out AST according to the modified Kirby Bauer method with 4 stages and 27 sub-stages (Table 1).

**TABLE 1:** Key indicators measured to check compliance of antimicrobial resistance sentinel site laboratories’ practices with Burkina Faso’s standardised national antimicrobial susceptibility testing procedure from 19 September 2022 to 28 September 2022 in Burkina Faso.

Sheet title	Key indicators
Laboratory performs quality control of culture media	To control or not the pH
	To measure or not the thickness of the culture medium
	Checking or not the fertility of the culture media
	Checking or not the sterility of the culture media
Laboratory performs quality control of antibiotic discs	Having or not *Escherichia coli* ATCC 25922 (CIP 7624)
	Having or not *Staphylococcus aureus* ATCC 25923 (CIP 7625)
	Having or not *Pseudomonas aeruginosa* ATCC 27853 (CIP 76110)
	Monitoring or not the condition of desiccants
	Defining or not the periodicity of quality control of the discs
	Number of quality controls carried out over a given period
	Verifying or not the diameters of the inhibition zones are checked
Analytical stages of carrying out AST according to the modified Kirby Bauer method	Preparation of the 0.5 McFarland turbidity standard
	Preparation of the inoculum
	Inoculation of plates, choice of antibiotic discs, arrangement of antibiotic discs
	Incubation and reading

AST, antimicrobial susceptibility testing.

### Evaluation team

Four teams evaluated the AMR sentinel sites laboratories from 19 September 2022 to 28 September 2022. Each team comprised two evaluators (a microbiologist and a quality manager with proven experience in medical biology) and a supervisor from the directorate of biomedical biology laboratories at the Ministry of Health. All the evaluation team members were trained to use the assessment tool appropriately to evaluate each activity.

### Evaluation method and period

The chosen approach was a two-day on-site observation of each sentinel laboratory and review of documents. The evaluation started with an introduction meeting followed by the questionnaire administration. As part of the process, the evaluation verified the existence of critical technical and administrative documents and essential points or steps of the pre-analytical, analytical and post-analytical phases.

The evaluation of AST practice focused on the following steps: preparation of the 0.5 McFarland bacterial inoculum, time and techniques for inoculating the bottles after preparing the bacterial suspension, choice and arrangement of antimicrobial discs, incubation times, temperature, and incubation condition after inoculation.

For the interpretative reading, the following aspects were evaluated: reading times after incubation, measurement of inhibition diameters, interpretation of tracer discs, and detection and reporting of phenotypes of interest for AMR monitoring. In the end, a debriefing meeting was held with the hospital management and laboratory staff to present the evaluation findings before the final report.

### Study variables

The records showing that activities to implement key quality control indicators for culture media and antibiotic discs were completed were checked to verify their compliance. Then, evaluators observed laboratory practices and assigned each quality indicator or stage a score. A stage completed per the requirements was worth 1 point. If the requirement was partially met, it was worth 0.5. A requirement not met was worth 0 points. All points obtained were used to calculate each laboratory’s compliance rate with the steps for carrying out the AST.

### Data analysis

Microsoft Excel software (Microsoft Corporation, Redmond, Washington, United States) was used for data entry and to produce graphs and tables. The free software R version 4.2.0^16^ was used to perform descriptive statistics. Score levels were defined to evaluate the overall performance of the laboratories. An overall score below 80% was considered noncompliance.^17^ A report resulting from the data analysis with corrective measures had been sent to the participating sentinel sites to improve their practices.

## Results

### Number and checking rate of culture media quality indicators

Regarding culture media quality indicators, 15 of 18 laboratories performed at least one quality control indicator (Table 2). All 15 laboratories tested the sterility and fertility of culture media, two laboratories checked media pH after preparation, and one measured the thickness of the agar.

**TABLE 2:** Number and rate of culture media quality indicators checked in antimicrobial resistance sentinel laboratories in Burkina Faso obtained through questioning from 19 September 2022 to 28 September 2022.

Indicator	Laboratories performing of culture media quality indicators (*N* = 15)	
	*n*	%
pH	2	13.33
Thickness	1	6.67
Fertility	15	100.00
Sterility	15	100.00

N, total number of laboratories controlling the quality of culture media; *n*, number of laboratories verifying the culture media quality indicator; %, percentage of laboratories verifying the quality indicators of the culture media.

### Checking rate of antibiotic disc quality indicators

A total of 12 of 18 laboratories performed at least one antibiotics disc quality control check (Table 3). Only 16.67% (*n* = 2 of 12) of the laboratories monitored the desiccant activity; 58.33% (*n* = 7 of 12) of the laboratories had defined periodic quality control check times for antibiotic discs, while 71.43% (*n* = 5 of 7) checked these quality indicators at the indicated frequency. In addition, 83.33% (*n* = 10 of 12) of the laboratories documented the inhibition diameters according to the critical diameters defined for the reference strain or antibiotic association.

**TABLE 3:** Number and rate of laboratories verifying the antibiotic discs’ quality indicators checked in antimicrobial resistance sentinel laboratories in Burkina Faso obtained through questioning from 19 September 2022 to 28 September 2022.

Indicator	Laboratories checking the quality indicators of discs (*N* = 12)	
	*n*	%
Desiccant activity	2	16.67
Determination of periodicity	7	58.33
Completion of QC at the indicated period	5	71.43†
Verification of the inhibition diameters	10	83.33

*N*, total number of laboratories controlling the quality of antibiotic discs; *n*, number of laboratories verifying the quality indicator of antibiotic discs; %, Percentage of laboratories verifying the indicator of antibiotic discs; QC, quality control.

† , (Completion of QC at the indicated period)/(Determination of periodicity).

### Availability of reference strains

**FIGURE 2:**
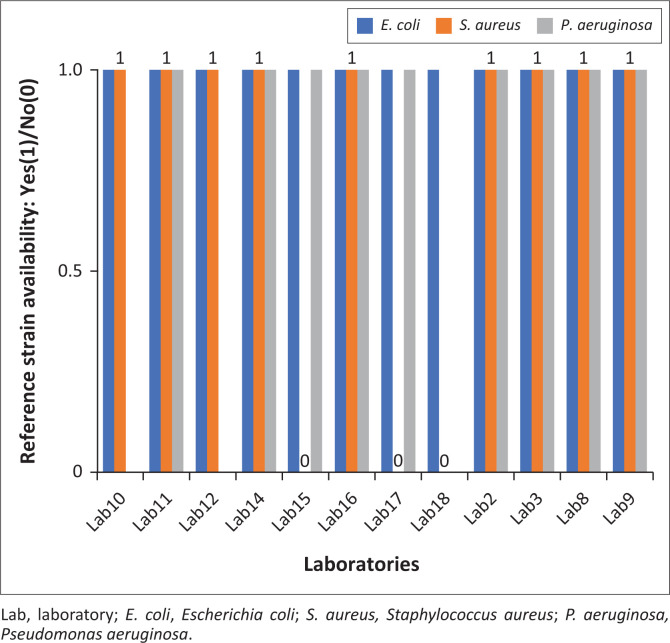
Availability of reference strains in the antimrobial resistance sentinel laboratories in Burkina Faso obtained through questioning from 19 September 2022 to 28 September 2022 in Burkina Faso.

All (*n* = 12 of 12) laboratories performing quality control of antibiotic discs had the reference strain *E. coli* ATCC 25922 (CIP 7624) (Figure 2). Two laboratories had the reference strains *of E. coli* ATCC 25922 (CIP 7624) and *S. aureus* ATCC 25923 (CIP 7625), and two laboratories had the reference strains of *E. coli* ATCC 25922 (CIP 7624) and *P. aeruginosa* ATCC 27853 (CIP 76110). Seven laboratories had all three reference strains.

### Compliance rate with modified Kirby Bauer method

**FIGURE 3:**
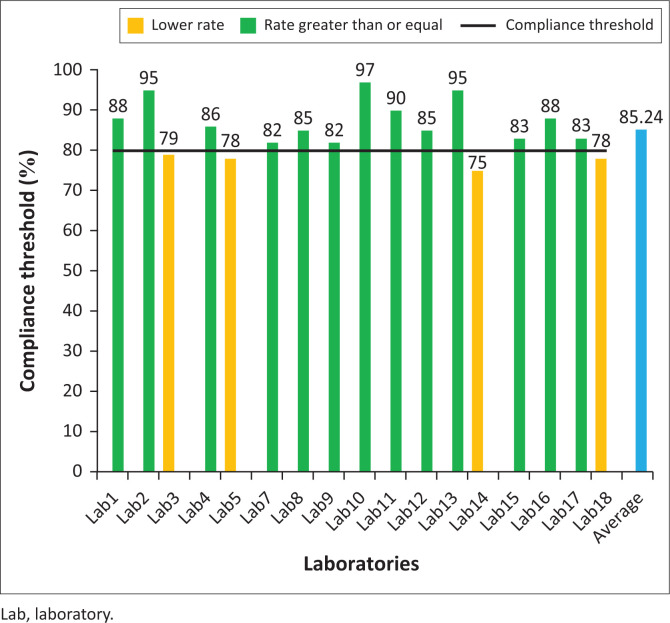
Compliance rate of antimicrobial resistance sentinel site laboratories in Burkina Faso with the modified Kirby Bauer method obtained through observation from 19 September 2022 to 28 September 2022 in Burkina Faso.

Most (*n* = 17 of 18) laboratories performed the routine ASTs according to the modified Kirby Bauer method described in the national procedure manual (Figure 3). The average compliance rate for all laboratories was 85.24%. The majority (*n* = 13 of 17) of laboratories had compliance rates above 80%. The highest and lowest rates obtained were 97% and 75%, respectively.

## Discussion

This study is the first to assess the implementation of quality indicators in the AMR sentinel site laboratories since Burkina Faso implemented the AMR surveillance in 2018. This activity has provided baseline data on the sentinel laboratories’ compliance with the national AST procedures manual, particularly in areas performing quality control checks or meeting quality indicators for culture media and antibiotic discs. The Ministry of Health meets the requirement to promote good practices in sentinel laboratories by conducting this assessment. An impartial review of documents and records and direct observation of practices allowed an objective assessment of compliance with the national procedure. We analysed the non-conformities identified during the evaluation to determine their impact and severity on operations, processes, patient outcomes, and safety considerations. Thus, the assessors proposed urgent actions depending on the criticality of each detected non-conformity. Each laboratory received reports with proposed improvement plans and recommendations.

A too-acidic agar pH increases ß-lactam activity, while an alkaline pH favours aminoglycoside and macrolide activity. Agar thickness has an impact on the diffusion gradient of the antibiotic.^18^ Nevertheless, a few laboratories controlled these parameters in our context. Maintaining the required agar thickness and pH depends on the medium dispensing technique, which requires a calibrated dispenser and a pH meter; however, only a few laboratories had these.

Fertility and sterility are the most important indicators in medium quality control. The medium must be carefully checked to avoid contamination before culture.^19^ All sentinel laboratories checked the culture medium quality indicators to ensure each medium met the microbiological characteristics before use.

Åhman et al. found that many discs exhibited significant quality issues, with 33% (*n* = 26 of 78) out-of-range readings in the first study and 17% (*n* = 20 of 120) in the second. Furthermore, discs from certain manufacturers showed unexpected variations in inhibition diameter zones (4 mm – 9 mm) for discs from the same vial.^20^ In our study, only 67% of our sentinel laboratories monitored the quality indicators of antibiotic discs; this situation could increase the chances of obtaining inaccurate results and, consequently, ineffective antibiotic therapy.^21^ This lack of disc quality control is concerning given the already alarming global resistance situation for some anitbiotics. Indeed, the resistance of *E. coli* to colistin, currently used as a last-resort antibiotic, has been observed in Asia, Europe, America, Africa, and Oceania (specific percentages for each continent: 66.72%, 25.49%, 5.19%, 2.27%, and 0.32%, respectively).^22,23^ The low rate of laboratories complying with these indicators can be attributed to staff mobility due to various assignments, lack of staff involvement, and the fact that, in most laboratories, personnel selection for training is not based on expressed training needs or competence assessments. In fact, in many laboratories, the staff trained in microbiological testing by the Ministry of Health and its partners did not necessarily come from the microbiology department due to an established internal rotation for various continuous training programmes. Staff were selected without necessarily considering the specific field covered by the training. We believe that the Ministry of Health should keep trained staff as long as possible. Furthermore, although participation in training is a source of motivation for staff, it would be good to take into account the specific field covered by the training in the selection of participants.

The reference strains are used to assess the overall performance of the test. They are used for quality control of all steps of culture and the study of bacterial sensitivity to antibiotics.^18^ Quality control of antibiotic discs using reference strains should be performed at least every two weeks.^12^ Performing checks at short intervals allows for a retrospective analysis of previous results to identify issues in the event of discrepant results, allowing for corrective actions to be taken in case of non-conformity results. Despite this, only 58.83% of the laboratories carrying out the quality controls of the antibiotic discs had defined periodicity of passage, and only 71.43% respected this predefined periodicity. This situation demonstrates that implementing the procedures in the national AST manual is still weak. The Ministry of Health should strengthen awareness, communication, and especially laboratory staff training.

### Limitations

The methodology used in our study did not verify the corrective measures taken in the event of non-conclusive control. Also, one laboratory was not evaluated for security reasons. Despite these methodological limitations, we obtained sufficient results that could be evaluated.

### Conclusion

The evaluation revealed non-conformities with the national AST procedure for carrying out AST at the sentinel laboratories level, particularly in the quality control component. We formulated proposals for improvement plans to address these non-conformities to provide reliable, accurate, and timely results, and to contribute effectively to the fight against AMR. Regular monitoring of quality indicators will ensure the reliability and accuracy of results from the sentinel sites laboratories.
